# Micromechanical Bolometers for Subterahertz Detection at Room Temperature

**DOI:** 10.1021/acsphotonics.1c01273

**Published:** 2022-01-28

**Authors:** Leonardo Vicarelli, Alessandro Tredicucci, Alessandro Pitanti

**Affiliations:** † Laboratorio NEST, Scuola Normale Superiore and Istituto Nanoscienze - CNR, Piazza San Silvestro 12, 56127 Pisa, Italy; ‡ Dipartimento di Fisica, Università di Pisa, Largo B. Pontecorvo 3, 56127 Pisa, Italy

**Keywords:** terahertz detection, bolometer, room temperature, self-mixing, micromechanical resonator, optomechanics

## Abstract

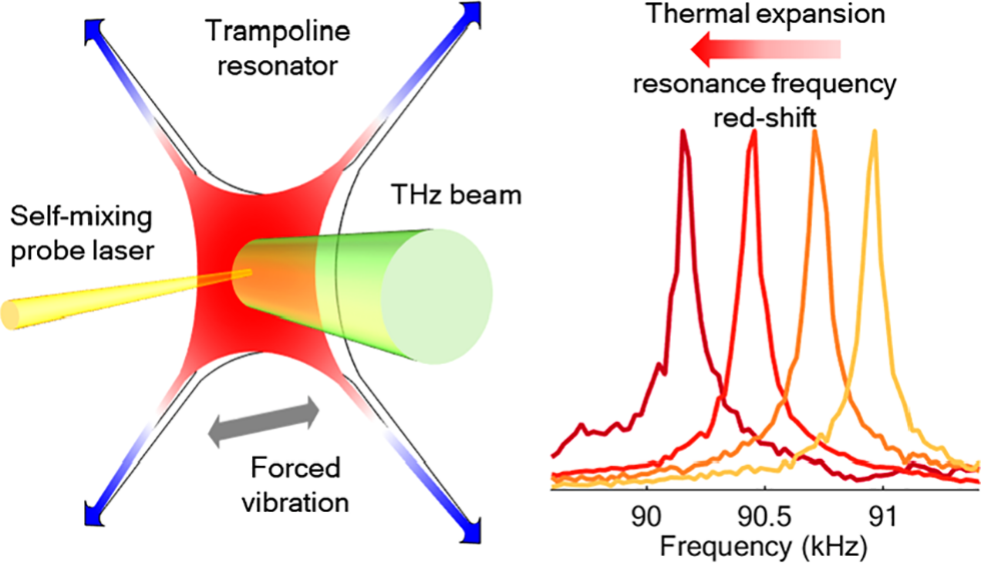

Fast room-temperature imaging at terahertz (THz) and subterahertz (sub-THz) frequencies is an interesting technique that could unleash the full potential of plenty of applications in security, healthcare, and industrial production. In this Letter, we introduce micromechanical bolometers based on silicon nitride trampoline membranes as broad-range detectors down to sub-THz frequencies. They show, at the longest wavelengths, room-temperature noise-equivalent powers comparable to those of state-of-the-art commercial devices (∼100 pW Hz^–1/2^), which, along with the good operation speed and the easy, large-scale fabrication process, could make the trampoline membrane the next candidate for cheap room-temperature THz imaging and related applications.

Terahertz (THz) radiation detectors have been developed for over a century,^1^ resulting in a large variety of solutions^2−4^ devised to suit an even larger range of applications, including astronomy,^5^ medical diagnostics,^6,7^ communications,^8^ industrial inspection,^9−11^ and security scanning of people^12^ and packages.^13^ However, broad exploitation of THz technology is still far from being achieved, as most of the proposed devices remain at the fundamental research level, unable to breach the commercialization barrier.^11,14^ Nevertheless, several user-friendly cameras^14,15^ for incoherent detection of THz (0.3–10 THz) and sub-THz (0.1–0.3 THz) radiation were recently introduced in the market with a relatively affordable price (a few thousand dollars). While R&D companies currently represent the majority of customers, a few of them were actually deployed in airports as THz body scanners,^16,17^ where noncontact security screening of passengers has become increasingly important. Despite the absence of spectroscopic information, an intensity-based THz image is in fact sufficient to identify dangerous items hidden under clothes, paper, or plastic^18^ without being harmful to human health.^19^ It therefore seems that a small market has formed, requiring inexpensive THz cameras with low-medium resolution (320 × 240 pixels) and operating at room temperature with video acquisition rate (30–60 Hz) and possibly with low noise (noise-equivalent power (NEP) < 100 pW Hz^–1/2^) in order to also passively detect human blackbody radiation.^20^

Among the most frequently adopted solutions for THz detection in these commercial cameras are microbolometer-based focal plane arrays (FPAs).^21−27^ Originally designed for the infrared range, they were adapted to detect THz radiation by tuning of the materials and structure of the absorption layer, addition of an antenna, and adjustment of the size of the resonant cavity. Such modifications led to impressive improvements in their detection capabilities, resulting in state-of-the-art low NEP (<10 pW Hz^–1/2^ and <500 pW Hz^–1/2^ above and below 1 THz, respectively^15^) and demonstrating that uncooled bolometers can be very competitive in this range. From this perspective, other bolometric approaches that were successful in the infrared range could in principle be modified to operate at lower frequencies.

Therefore, we have focused our attention on an emerging type of bolometric detector based on microelectromechanical (MEMS) resonators. In this kind of device, a small suspended structure (typical size 10–1000 μm) can freely vibrate in air or vacuum at its own characteristic resonance frequency, which can be measured by electrical or optical means. The heat generated by the absorbed radiation induces a thermal expansion of the vibrating structure, shifting its resonance frequency. Considering exclusively the infrared spectral range, several MEMS resonator bolometers have been proposed in recent years, resulting in a wide assortment of geometries and materials. These include GaN plates suspended by two tethers,^28^ Si_3_N_4_ drums with a thin-film absorber,^29^ a Si torsional resonator with a TiN absorber,^30,31^ graphene–AlN–Pt nanoplates,^32^ SiN phononic crystal membranes,^33^ and graphene drum/trampolines.^34^ To date and limited to our knowledge, only one work has demonstrated a MEMS resonator capable of room-temperature bolometric detection in the THz range.^35,36^ That device, based on doubly clamped GaAs beams coated with a 15 nm-thick NiCr THz absorbing film, exhibited good performance in terms of noise (optical NEP ≈ 500 pW Hz^–1/2^) and speed (1 kHz bandwidth) at room temperature with the aid of a Si hemispherical lens. Another MEMS approach based on GaAs–Au meta-atom THz resonators^37,38^ exploits combined Coulomb/photothermal detection; the fast device operation achieved (MHz) relies on radiation source modulation at the mechanical mode frequency.

In this work, we show the realization of an ultrasensitive micromechanical resonator bolometer using a Si_3_N_4_ trampoline with a 35 nm-thick Cr–Au coating, challenging the state of the art for room-temperature bolometric detectors in the sub-THz range (140 GHz) with a minimum NEP of ∼100 pW Hz^–1/2^ and a detection speed of 40 Hz. Additionally, we demonstrate broadband bolometric detection covering both infrared and visible light.

The nontrivial choice of the Si_3_N_4_ trampoline as the most suitable platform for our bolometric detector is the result of an extensive analysis. The Si_3_N_4_ trampoline has excelled in many ultrasensitive applications, such as electron spin detection,^39^ position sensors,^40^ and displacement detectors^41,42^ with picometer resolution. In contrast to microstring/plate/beam MEMS resonators, the size of the trampoline membrane can be scaled to match the diffraction-limited area at THz frequencies, thus maximizing radiation absorption. At the same time, the small width of the tethers minimizes heat losses to the surrounding Si frame, ensuring the formation of a steep temperature gradient. Compared with the conceptually similar graphene trampoline,^34^ our device performs worse in the infrared range but offers a much simpler, economical, and well-established fabrication procedure that can easily be extended to larger scales for the realization of arrays and possibly inexpensive multipixel THz cameras.

## Experimental Setup

**Figure 1:**
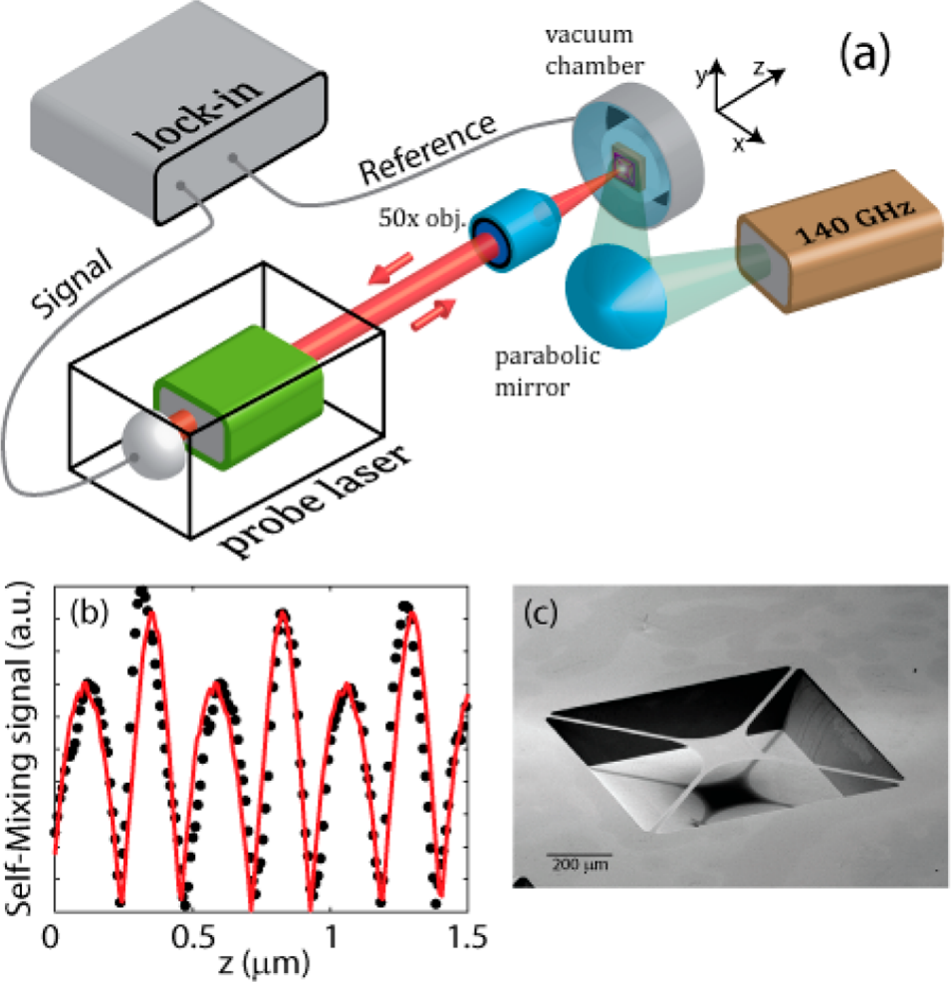
(a) Sketch of the experimental setup. (b) Experimental (black dots) and numerical (red line) static self-mixing signals. (c) SEM micrograph of a trampoline membrane (device M2).

The setup we used to characterize our device is sketched in Figure 1a. Similar to other MEMS-based bolometric approaches^31,33,37^ and Golay cells,^43^ we performed the readout of the trampoline vibrational frequency by optical means. We used a self-mixing (SM) interferometric technique with a near-infrared laser (945 nm) working as a probe; after emission and focusing on the trampoline’s surface, the laser is reflected back into the cavity itself. Fluctuations of the intracavity field amplitude carry the memory of light interacting with the environment and therefore can be used to probe tiny movement of the trampoline. In our setup, this can be done using a photodiode integrated within the laser cavity to probe the laser power. The dynamics of SM is well-described through the Lang–Kobayashi equations,^44^ which under steady-state conditions can be reduced to compact expressions for the laser emission frequency ω_SM_, the cavity carrier density *n*_s_, and the photon density *P*_s_:^45^
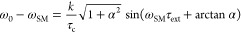
1
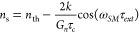
2
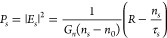
3where ω_0_ is the laser frequency without feedback, α is the linewidth enhancement factor, *k* is a matching constant, *G*_*n*_ is the modal gain factor, *R* the carrier injection rate, *n*_th_ and *n*_0_ are the carrier densities at threshold and transparency, respectively, and τ_c_, τ_s_, and τ_ext_ are the cavity round-trip time, the carrier lifetime, and the feedback time, respectively. The last of these is the time necessary for the photons to return into the cavity after emission. A small vibration of the reflecting target translates into a change in τ_ext_, changing the solution of eqs 1–3. In a preliminary characterization experiment, as a target we employed a fixed mirror mounted on a piezoelectric actuator and on a motorized stage. Using a lock-in amplifier, we applied a reference sinusoidal voltage to the piezoelectric actuator to induce small oscillations (a few tens of nm) of the mirror along the *z* axis and then acquired the demodulated SM signal at different distances (*z* axis) from the laser itself, moving the motorized stage. The resulting signal amplitude is shown as black dots in Figure 1b. The experimental data are in good agreement with numerical analysis based on the aforementioned equations, where the parameters used are compatible with values reported in the literature.^41^ After calibration, we replaced the fixed mirror with the Si_3_N_4_ trampoline microresonators.

We fabricated and characterized two different membranes starting from a 300 nm-thick Si_3_N_4_ film: the first one (M1) has a 300 μm × 300 μm central plate and 490 μm × 20 μm tethers, while the second (M2) has a smaller 200 μm × 200 μm plate and longer 560 μm × 20 μm tethers. Both membranes were clamped to a 1 mm × 1 mm square Si supporting frame. A scanning electron microscopy (SEM) micrograph of device M2 is shown in Figure 1 (c). We metalized both resonators with 5/30 nm Cr/Au in order to increase the material absorption and the overall thermal conductivity. More details on the device fabrication are reported in Methods, and the mechanical, thermal, and optical properties of the metallic and Si_3_N_4_ layers are given in Tables S1–S3.

## Infrared Bolometry

**Figure 2:**
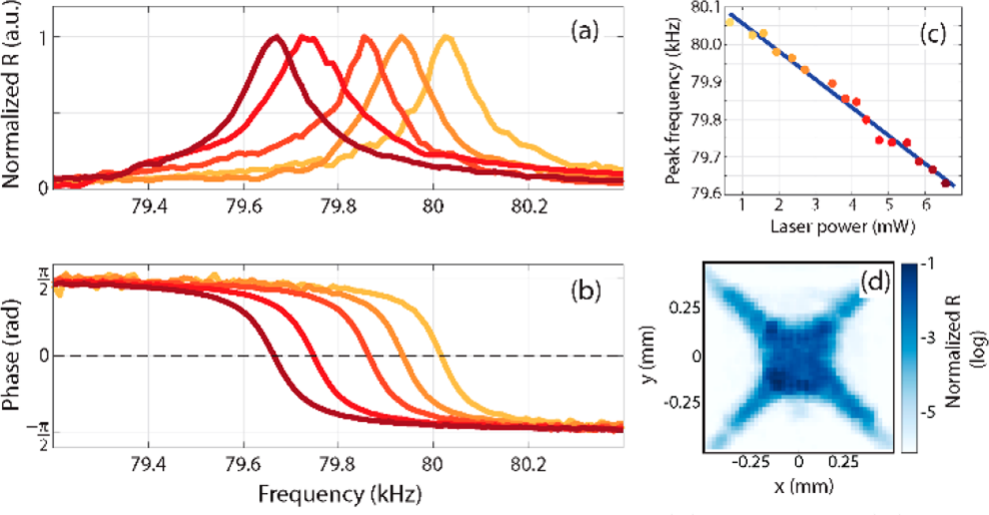
Device M1. (a) Demodulated amplitude and (b) phase when the trampoline resonator was probed at different laser powers. (c) Shift of the resonant frequency as a function of laser power. (d) Map of the demodulated amplitude around the membrane center.

Starting from device M1, we placed the microresonator on a piezoelectric actuator inside a vacuum chamber with a transparent window in order to grant light access (visible, infrared, and THz). We shined the laser light at the trampoline center and used a sinusoidal voltage as the bias for the piezo actuator as well as the reference for the lock-in demodulation, similar to the fixed mirror configuration. When the voltage frequency was resonant with one of the membrane modes, a clear signal appeared in the lock-in amplifier, as can be seen in Figure 2a. The demodulated amplitude has a Lorentzian shape with a corresponding phase slip of π when crossing the resonance frequency, located approximately at 80 kHz for this particular membrane. We identified this mode as the fundamental drum mode of the mechanical resonator, which is characterized by a rigid shift of the central plate perpendicular to the membrane plane. The map shown in Figure 2d agrees with our observation, showing a constant demodulated signal across the membrane center with a decreasing signal running around the tethers. We estimated the quality factor of this mode to be *Q* ≈ 900 from best-fit analysis of the Lorentzian curve.

Interestingly, the resonance peak position depends linearly on the impinging power of the SM infrared laser. As can be seen in Figure 2c as well as in the set of curves in panels (a) and (b), increasing laser power red-shifts the resonance with a slope of 75 kHz/W, corresponding to a normalized responsivity of 0.94 W^–1^ (calculated using the resonance frequency at zero incident power, *f*_0_ = 80.1 kHz). This is due to an increase in the device temperature induced by the laser heating.

In order to explore the dependence of the responsivity on the trampoline geometry, we repeated the characterization on device M2. As the beam spot size of the infrared laser is considerably smaller (36 μm) than the central plate size (200–300 μm), the amount of absorbed radiation is the same for both M1 and M2, but the latter has longer tethers, resulting in a lower thermal conductance and therefore a higher responsivity. This observation was indeed confirmed by the measured responsivity of 187 kHz/W for device M2, resulting in a normalized responsivity of 2.05 W^–1^ (Figure S1). For this particular device, we also quantified the contribution of the Cr/Au layer to the absorption of the infrared light: the measured responsivity before the Cr/Au layer deposition was merely 8 kHz/W, approximately 23 times less than the 187 kHz/W value mentioned earlier (see Figure S2). This confirms the importance of an appropriate choice for the coating of the Si_3_N_4_ structure, which would otherwise be mostly transparent in this region of the electromagnetic spectrum.^46^

To better understand the observed responsivity values, we performed finite element method (FEM) simulations of the two devices and compared the results (see Methods for details about the simulation). The simulated infrared responsivities were 560 and 620 kHz/W for devices M1 and M2, respectively, in qualitative agreement with the measured values. The fact that the simulated values are 3 to 7 times higher than the measured ones could be attributed to small defects in the actual membrane geometry and uncertainties in the thin Si_3_N_4_ and Cr/Au film empirical parameters, such as thermal conductivity, intrinsic stress, and thermal expansion coefficient, which are strongly dependent on the thickness (or the Si content for Si_3_N_4_) and can be very different from the bulk values.^47−50^

## Broadband Characterization

After this initial characterization in the infrared range, we continued exploring the bolometric effect of our thermomechanical device using an external illumination source while keeping the probe laser as weak as possible (1.5 mW, slightly above the lasing threshold). To show the broadband operation of our device, we used a green laser diode (563 THz, or 532 nm) and a sub-THz microwave source (0.14 THz). Both sources were mounted following the scheme of Figure 1a, employing a parabolic mirror to focus the emission on the trampoline membrane. Despite the focusing, the spot size of the sub-THz beam in the detector plane was approximately 7.1 mm × 4.3 mm (see Figure S5), which is significantly larger than the device. For this reason, the sub-THz responsivities were calculated using the incident power inside the diffraction-limited area λ^2^/4 ≈ 1.15 mm^2^, which is equal to 0.32 mW. To be more specific about this choice, we did not use the active area of the device (0.15 mm^2^ for device M1, equal to the surface of the central plane plus tethers) to calculate the incident power because that is much smaller than the diffraction-limited area achievable at 0.14 THz. Instead, the green laser was entirely focused inside the trampoline’s central plate (spot size = 108 μm; see Figure S6), but its intensity was reduced with a 2.1 neutral density (ND) filter, resulting in a total incident power of 0.016 mW.

In order to measure the responsivities corresponding to these external sources, we modified the detection experiment to operate in an open-loop configuration, fixing the excitation frequency of the piezo actuator and monitoring the temperature-induced phase shift of the demodulated signal. As visual guidance for the reader, this corresponds to the point of maximum slope in Figure 2b.

**Figure 3:**
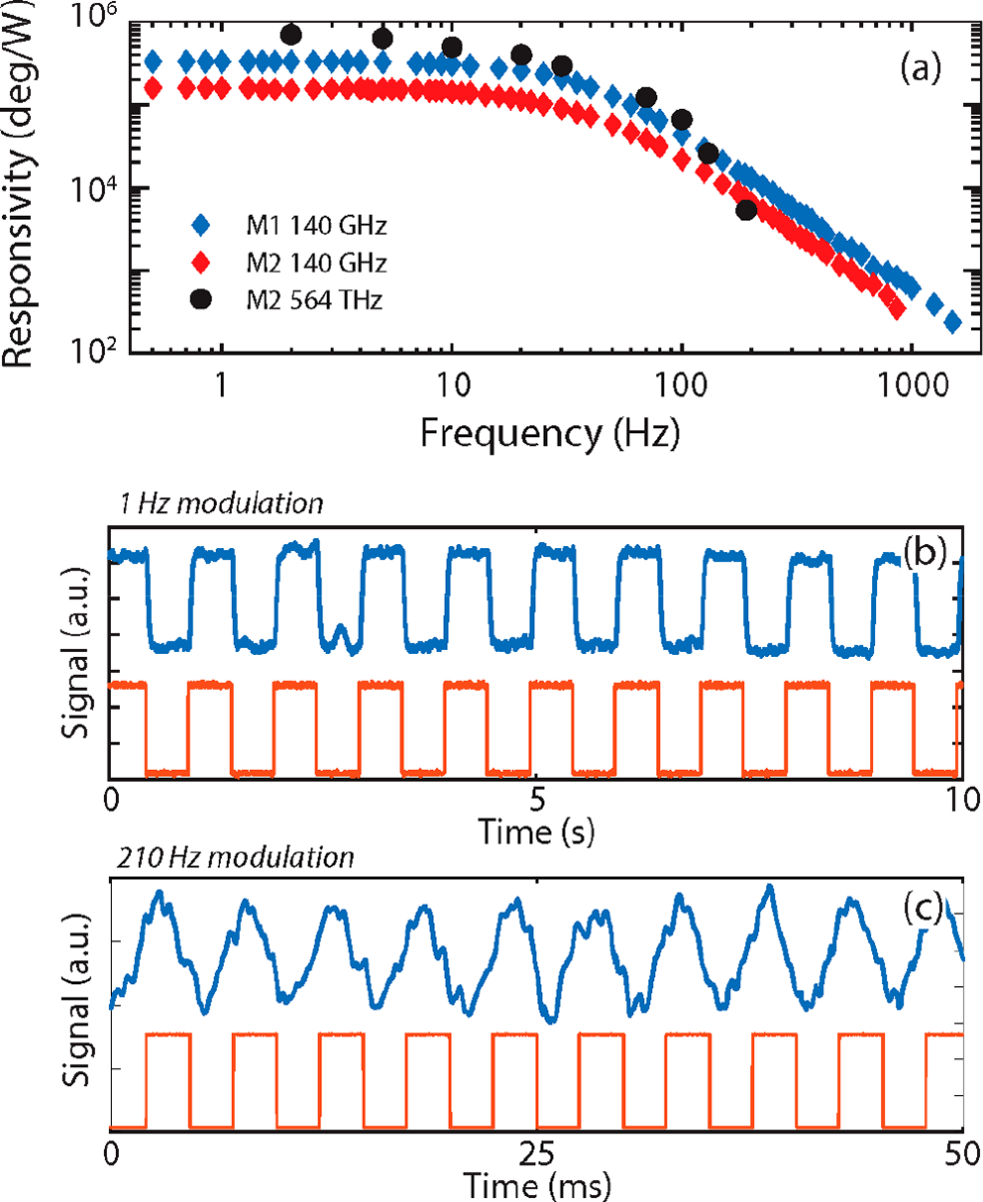
(a) Responsivities of the two different membranes as functions of the modulation frequency for visible (564 THz) and sub-THz (0.14 THz) radiation. (b, c) Time plots of the phase signal for membrane M1 illuminated by the 0.14 THz source for modulation frequencies of 1 and 210 Hz, respectively (blue = signal; orange = TTL modulation)

The phase responsivities measured on the two membranes, employing both sources, are reported in Figure 3a. The measurements were performed with modulated sources in order to assess the operational speed of the thermomechanical bolometers. The maximum values, reached at low modulation frequencies, are 350 kHz/W (4.4 W^–1^ normalized) and 140 kHz/W (1.6 W^–1^ normalized) with the sub-THz source for membranes M1 and M2, respectively, while the responsivity for the green laser reaches a maximum at 560 kHz/W (6.2 W^–1^ normalized) on device M2. The green-laser responsivities of both devices were also measured at zero modulation frequency in a similar configuration to the infrared case, tuning the power of the green laser with ND filters of increasing opacity. The measured responsivities are 520 and 600 kHz/W for devices M1 and M2, respectively, in agreement with the values obtained by looking at the phase variation (the measurements are reported in Figures S3 and S4). With regard to the modulation frequency dependence, the two devices show qualitatively similar trends, with 3 dB cutoff frequencies of about 40 and 20 Hz for the sub-THz and visible sources, respectively. The bottleneck here is represented by the thermal dynamics, which is governed by the dissipation of the membrane through the tethers. This is also confirmed by the thermal response frequency of the devices, which is calculated as the ratio between the thermal conductance of the tethers and the heat capacity of the central plate and is approximately equal to 10–20 Hz (see Table S3). By changing the tether geometry and material composition, one can expect to be able to increase the dissipation and device operational speed at the expense of a possible reduction in the responsivity. The presence of the Cr/Au metallic coating in our devices is particularly relevant to this end, as it contributes more than 80% of the total thermal conductance (see Table S3). The slight difference in the results from the two sources illuminating device M2 can be ascribed to the very different spot sizes, as described above. In fact, the broad illumination from the sub-THz source implies that the incident power is dissipated faster, having a shorter average path to the thermal heat sink.

Another consequence of the larger illumination area is that the sub-THz responsivity of device M1 is always higher than that of device M2, opposite to what we observed with the infrared laser. Here the straightforward explanation comes from the larger surface are of device M1 with respect to M2, implying a higher total absorbed radiation. To be more specific, the approximate ratio of 2.2 between the areas of the central plates is compatible with the ratio of 2.5 between the measured responsivities. Examples of detected signals in the time domain are reported in Figure 3b,c for device M1 illuminated at 0.14 THz. As can be seen, the detected signal (blue traces) perfectly reproduces the modulated source input (orange traces) for 1 Hz modulation; despite the presence of some deformations at 210 Hz modulation, the detected signal clearly follows the source dynamics.

We repeated the FEM simulations with the sub-THz source and obtained responsivities of 200 kHz/W (M1) and 130 kHz/W (M2), close to the measured absolute values but with a ratio of 1.5, which is smaller than the experimental one. Lastly, the simulated green-laser responsivity resulted in a much higher value of 2700 kHz/W (M2) because of the combination of higher absorption (17%; see Table S2) and more focused beam.

Comparing the detection configuration used earlier with the infrared source (i.e., frequency shift) against the one adopted for external sources (i.e., phase change) shows that the main advantage of the latter is a much higher detection speed, which is not limited by the time required to acquire a full frequency sweep but rather by the physical limit of the device. Moreover, in the phase detection scheme, the importance of the microresonator quality factor is particularly evident, since it both defines the sensitivity and limits the maximum detectable power, defined by the small range where the phase variation is linear near the resonance peak. A very high quality factor^39^ (*Q* > 10^6^) would lead to higher sensitivities but would result in a drawback given by a reduced power operating range. Our devices, in particular, with a measured *Q* of ∼900, give a good compromise between sensitivity and operating range. If we consider the phase range from −π/3 to π/3 acceptable for phase detection, it translates approximately into a 150 Hz frequency-shift range for device M1, corresponding to a maximum detectable power of 0.43 mW for the sub-THz source. With the minimum NEP (discussed in the next section) at a detection speed of 10 Hz, which is on the order of 0.3 nW, this gives a dynamic range of approximately 60 dB. For higher power, one could still operate the device in the frequency-shift detection scheme, thus further increasing the dynamic range.

## Noise Measurements

**Figure 4:**
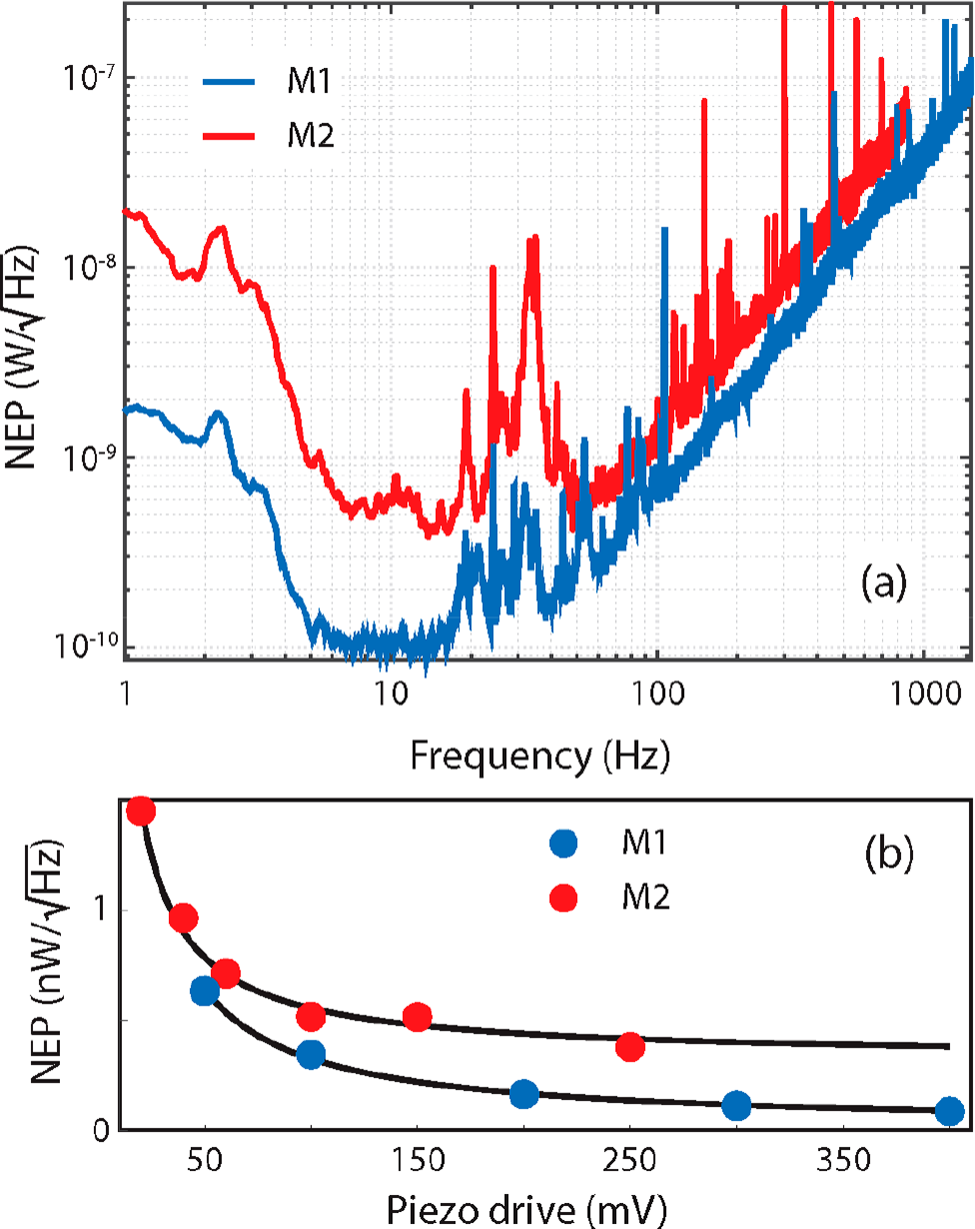
(a) NEPs of the two membranes illuminated by the 0.14 THz source as functions of the modulation frequency. (b) Minimum NEPs as functions of the amplitude of the sinusoidal driving voltage applied to the piezoelectric actuator.

To better estimate the thermomechanical bolometer performances, we considered the noise-equivalent power, which represents the incident power producing a detected signal-to-noise ratio of 1. This was obtained by taking the ratio of the measured spectral noise density and the responsivity reported in Figure 3a. Figure 4 shows the NEPs of devices M1 and M2 as functions of the modulation frequency with the 0.14 THz source. The two devices share a similar trend, but device M1 consistently shows a lower NEP compared with device M2 thanks to its higher responsivity.

In the 7–14 Hz modulation interval, where the curve is mostly flat, our best device reached a minimum NEP of ∼100 pW Hz^–1/2^. For detectors operating at room temperature in the THz and sub-THz ranges, this NEP value is competitive with those for the state-of-the-art technology, represented by vanadium oxide microbolometers,^21−25^ Schottky diodes,^51^ NMOS detectors,^52,53^ Dyakonov–Shur plasma wave detectors,^54−57^ and many other photodetectors based on 2D crystals.^58−62^ Below 7 Hz modulation, the NEP rapidly increases because of a combination of 1/*f* noise, thermal drift,^63−65^ and adsorption–desorption noise.^65^ Instabilities of the SM optical readout also contribute to the low-frequency noise. In fact, air flow and small drifts of the various optical elements will change the optical path length, inducing occasional mode hopping in the LD.^66^ At higher frequencies, the NEP increases above 14 Hz because of the declining responsivity, while the noise remains white. On top of this general trend, several resonance peaks are visible across the whole spectrum that are related to environmental vibrations.

The NEP was also found to be strongly dependent on the amplitude of the membrane’s vibration, which can be adjusted via the sinusoidal driving voltage sent to the piezoelectric actuator. In particular, the minimum NEP (in the 7–14 Hz region) is inversely proportional to the driving voltage, as shown in Figure 4b, and tends to decrease at higher voltages. This behavior is related to the fact that the SM signal and therefore the vibration amplitude both increase linearly with the driving voltage (see Figure S7) while the noise remains constant. This trend remains valid until the vibration amplitude saturates and nonlinear effects come into play, which in our case occurs above the piezo driving voltage of 450 mV.

Interestingly, the piezo drive represents the main source of power needed to operate the device. Considering a 150 mV drive, which gives an NEP of ∼200 pW Hz^–1/2^, a single thermomechanical bolometer dissipates a power of 225 nW. This relates well to the possibilities of creating a grid of resonators that can be used for sub-THz imaging and similar applications.

## Conclusion and Outlook

We have shown how Si_3_N_4_ trampoline membrane resonators can be an optimal platform for thermomechanical bolometric detection at room temperature over a broad frequency range down to sub-THz frequencies. Direct detection experiments have shown NEPs comparable with those of state-of-the-art commercial devices. To increase the device responsivity in the THz range, absorption could be enhanced by tailoring the surface of the trampoline resonator with different materials and geometrical patterns. For example, vanadium oxide metasurfaces^67^ and metal–dielectric–metal structures^68^ have been reported to absorb more than 90% of radiation around 1 THz. The type of absorption layer should, in general, be carefully evaluated, as it might introduce additional mechanical losses, which should be minimized to achieve higher quality factors, thus increasing the detection sensitivity. In order to implement an array detection scheme based on optical readout, a compact structure could be devised following the example of Tucker et al.,^69^ who illuminated the trampoline from the back side with the self-mixing laser and from the front side with the THz beam. Both sides of the trampoline could be easily accessed with complete etching of the Si substrate by extending the duration of the KOH etching. Alternatively, an electronic readout based on a magnetomotive force^40^ actuator/sensor could be introduced. These improvements, together with the simple and well-established fabrication procedures, would pave the way for large-scale fabrication of arrays of detectors, making the investigated platform extremely appealing for THz and sub-THz spatially resolved detection and cameras.

## Methods

### Sample Fabrication

The trampolines were fabricated starting from a 300 nm thick, high-stress (∼900 MPa), stoichiometric LPCVD Si_3_N_4_ film grown on top of a 250 μm thick Si wafer. The trampoline shape was patterned with optical lithography (DMO MicroWriter ML3), carefully smoothing all sharp corners to reduce stress at critical points such as where the tethers are clamped to the Si frame. The unmasked Si_3_N_4_ was removed by a reactive ion etching (RIE) step with a CF_4_/H_2_ gas mixture. Then the trampolines were released with hot KOH etching solution (30% concentration). Finally, a 5/30 nm Cr/Au metallic layer was thermally evaporated on top of the whole device surface.

### Experimental Setup

The positions of the various elements in the setup are shown in Figure 1. The Si chip containing the Si_3_N_4_ membranes was mounted inside a small vacuum chamber (2 × 10^–3^ mbar) and sealed with a cyclic olefin copolymer (COC) window that was transparent to both visible and THz radiation (87.7% transmission in the infrared and visible range, 80% in the sub-THz range). The chamber was actively pumped for the whole duration of the experiment using a turbomolecular pump, and the pressure was constantly monitored with a compact cold cathode gauge. The whole chamber could be moved with step motors in the *x*–*y* plane, orthogonal to the optical beam axis. Inside the chamber, the Si chip was glued on top of a piezoelectric actuator (used to excite the membrane vibrations) that was then attached with double-sided tape to the chamber itself. A Littrow external cavity diode laser (ECDL) (Toptica model DL100-L, 945 nm wavelength) was used to sense the membrane displacement via the self-mixing interferometric technique. The ECDL beam was focused on the membrane with an objective lens (Mitutoyo M Plan Apo 10×/0.28, 200 mm focal length). The output of the photodiode mounted on the back-side facet of the ECDL, which measured the fluctuations of the laser intensity due to self-mixing, was sent to a lock-in amplifier (Zurich Instruments MFLI). The Lorentzian curves shown in Figure 2a were generated with sequential frequency sweeps of the sinusoidal voltage sent to the piezoelectric actuator, using only one channel of the lock-in amplifier.

The radiation generated by the green laser and sub-THz source was focused on the membrane using an off-axis parabolic mirror (gold-coated, 2″ focal length). The 0.14 THz beam was generated by a commercially available source (TeraSense Group) based on an impact ionization avalanche transit time (IMPATT) diode equipped with a conical horn antenna. The 564 THz (λ = 532 nm) source was a commercial green laser pointer attenuated with an additional 2.1 ND filter in order to keep a lower incident power on the trampoline. With a calibrated pyrometer, we measured the output powers of both sources after the reflection in the parabolic mirror and the COC window, resulting in 10.6 mW for the sub-THz beam and 2.0 mW for the green laser, the latter of which was reduced to 0.016 mW after the ND filter. The spot sizes of the infrared ECDL beam and the green laser were measured with the knife-edge method, as illustrated in Figure S6. The spot size of the sub-THz beam was measured by mapping the illuminated area in the sample plane with the pyrometer, as shown in Figure S5.

In order to measure the responsivity of the membrane detector as a function of the source modulation frequency, a mechanical chopper (maximum 200 Hz rotation) and a TTL signal were used for the green laser and the sub-THz source, respectively. The responsivity, expressed in degrees per watt, was measured using two separate lock-in amplifiers (Zurich Instruments MFLI and Stanford Research SR830) in cascade. The membrane was excited at a fixed frequency and set at the middle point between the resonances measured with and without radiation, and the phase output of the first lock-in amplifier was sent to a second lock-in amplifier, which used the chopper or TTL as a reference.

### FEM Simulations

We performed the FEM simulations with COMSOL Multiphysics version 5.5. The 2D trampoline shape was meshed with a free triangular mesh and then swept vertically to mesh the 3D structure. The Cr/Au layer was simulated as a single 35 nm thick Au element. The mechanics and heating were simulated simultaneously using the “thermal expansion” multiphysics coupling. The heating due to the lasers and THz source was simulated with the “Deposited Beam Power” option using a Gaussian beam profile. A “time-dependent” study simulated the initial temperature spatial profile, followed by a “pre-stressed eigenfrequency” study to extract the frequency of the fundamental drum mode. The material parameters used in the simulation for Si_3_N_4_ and Au are listed in Tables S1 and S2.

## Supporting Information Available

List of parameters used in COMSOL simulations, infrared responsivity of device M22 before and after Cr/Au deposition, green laser responsivities of devices M1 and M2, spot size measurements of green, infrared, and sub-THz sources, and dependence of the self-mixing signal as a function of the piezo driving voltage amplitude (PDF)

## References

[ref1] Sizov F. F. Brief History of THz and IR Technologies. Semicond. Phys., Quantum Electron. Optoelectron. 2019, 22 (1), 67–79. 10.15407/spqeo22.01.067.

[ref2] Lewis R. A. A Review of Terahertz Detectors. J. Phys. D. Appl. Phys. 2019, 52 (43), 433001 10.1088/1361-6463/ab31d5.

[ref3] Sizov F. Terahertz Radiation Detectors: The State-of-the-Art. Semicond. Sci. Technol. 2018, 33 (12), 123001 10.1088/1361-6641/aae473.

[ref4] Dhillon S. S.; Vitiello M. S.; Linfield E. H.; Davies A. G.; Hoffmann M. C.; Booske J.; Paoloni C.; Gensch M.; Weightman P.; Williams G. P.; Castro-Camus E.; Cumming D. R. S.; Simoens F.; Escorcia-Carranza I.; Grant J.; Lucyszyn S.; Kuwata-Gonokami M.; Konishi K.; Koch M.; Schmuttenmaer C. A.; Cocker T. L.; Huber R.; Markelz A. G.; Taylor Z. D.; Wallace V. P.; Axel Zeitler J.; Sibik J.; Korter T. M.; Ellison B.; Rea S.; Goldsmith P.; Cooper K. B.; Appleby R.; Pardo D.; Huggard P. G.; Krozer V.; Shams H.; Fice M.; Renaud C.; Seeds A.; Stöhr A.; Naftaly M.; Ridler N.; Clarke R.; Cunningham J. E.; Johnston M. B. The 2017 Terahertz Science and Technology Roadmap. J. Phys. D. Appl. Phys. 2017, 50 (4), 043001 10.1088/1361-6463/50/4/043001.

[ref5] Walker C. K. Terahertz Astronomy; CRC Press, 2016.

[ref6] Yu L.; Hao L.; Meiqiong T.; Jiaoqi H.; Wei L.; Jinying D.; Xueping C.; Weiling F.; Yang Z. The Medical Application of Terahertz Technology in Non-Invasive Detection of Cells and Tissues: Opportunities and Challenges. RSC Adv. 2019, 9 (17), 9354–9363. 10.1039/C8RA10605C. PMC9062338 35520739

[ref7] Zaytsev K. I.; Dolganova I. N.; Chernomyrdin N. V.; Katyba G. M.; Gavdush A. A.; Cherkasova O. P.; Komandin G. A.; Shchedrina M. A.; Khodan A. N.; Ponomarev D. S.; Reshetov I. V.; Karasik V. E.; Skorobogatiy M.; Kurlov V. N.; Tuchin V. V. The Progress and Perspectives of Terahertz Technology for Diagnosis of Neoplasms: A Review. J. Opt. 2020, 22 (1), 013001 10.1088/2040-8986/ab4dc3.

[ref8] Koenig S.; Lopez-Diaz D.; Antes J.; Boes F.; Henneberger R.; Leuther A.; Tessmann A.; Schmogrow R.; Hillerkuss D.; Palmer R.; Zwick T.; Koos C.; Freude W.; Ambacher O.; Leuthold J.; Kallfass I. Wireless Sub-THz Communication System with High Data Rate. Nat. Photonics 2013, 7 (12), 977–981. 10.1038/nphoton.2013.275.

[ref9] Stecher M.; Jördens C.; Krumbholz N.; Jansen C.; Scheller M.; Wilk R.; Peters O.; Scherger B.; Ewers B.; Koch M. Towards Industrial Inspection with THz Systems. Springer Ser. Opt. Sci. 2016, 195, 311–335. 10.1007/978-3-319-17659-8_14.

[ref10] Tao Y. H.; Fitzgerald A. J.; Wallace V. P. Non-Contact, Non-Destructive Testing in Various Industrial Sectors with Terahertz Technology. Sensors 2020, 20 (3), 712 10.3390/s20030712. PMC7039230 32012901

[ref11] Naftaly M.; Vieweg N.; Deninger A. Industrial Applications of Terahertz Sensing: State of Play. Sensors 2019, 19 (19), 4203 10.3390/s19194203. PMC6806174 31569789

[ref12] Tzydynzhapov G.; Gusikhin P.; Muravev V.; Dremin A.; Nefyodov Y.; Kukushkin I. New Real-Time Sub-Terahertz Security Body Scanner. J. Infrared, Millimeter, Terahertz Waves 2020, 41 (6), 632–641. 10.1007/s10762-020-00683-5.

[ref13] Shchepetilnikov A. V.; Gusikhin P. A.; Muravev V. M.; Tsydynzhapov G. E.; Nefyodov Y. A.; Dremin A. A.; Kukushkin I. V. New Ultra-Fast Sub-Terahertz Linear Scanner for Postal Security Screening. J. Infrared, Millimeter, Terahertz Waves 2020, 41 (6), 655–664. 10.1007/s10762-020-00692-4.

[ref14] Simoens F. Buyer’s Guide for a Terahertz (THz) Camera. Photoniques 2018, (Special EOS Issue 2), 58–62.

[ref15] Oda N. Technology Trend in Real-Time, Uncooled Image Sensors for Sub-THz and THz Wave Detection. Proc. SPIE 2016, 9836, 98362P 10.1117/12.2222290.

[ref16] MC2 Technologies. MM-Imager. https://www.mc2-technologies.com/mm-imager (accessed 2022-01-10).

[ref17] Thruvision. Customs and border security cameras. https://thruvision.com/products/customs-and-border-security-cameras (accessed 2022-01-10).

[ref18] Federici J. F.; Schulkin B.; Huang F.; Gary D.; Barat R.; Oliveira F.; Zimdars D. THz Imaging and Sensing for Security Applications—Explosives, Weapons and Drugs. Semicond. Sci. Technol. 2005, 20 (7), S266–S280. 10.1088/0268-1242/20/7/018.

[ref19] Kleine-Ostmann T.; Jastrow C.; Baaske K.; Heinen B.; Schwerdtfeger M.; Karst U.; Hintzsche H.; Stopper H.; Koch M.; Schrader T. Field Exposure and Dosimetry in the THz Frequency Range. IEEE Trans. Terahertz Sci. Technol. 2014, 4 (1), 12–25. 10.1109/TTHZ.2013.2293115.

[ref20] Čibiraitė-Lukenskienė D.; Ikamas K.; Lisauskas T.; Krozer V.; Roskos H. G.; Lisauskas A. Passive Detection and Imaging of Human Body Radiation Using an Uncooled Field-Effect Transistor-Based THz Detector. Sensors 2020, 20 (15), 4087 10.3390/s20154087. PMC7435787 32707924

[ref21] Oda N.; Kurashina S.; Miyoshi M.; Doi K.; Ishi T.; Sudou T.; Morimoto T.; Goto H.; Sasaki T. Microbolometer Terahertz Focal Plane Array and Camera with Improved Sensitivity in the Sub-Terahertz Region. J. Infrared, Millimeter, Terahertz Waves 2015, 36 (10), 947–960. 10.1007/s10762-015-0184-2.

[ref22] Dufour D.; Marchese L.; Terroux M.; Oulachgar H.; Généreux F.; Doucet M.; Mercier L.; Tremblay B.; Alain C.; Beaupré P.; Blanchard N.; Bolduc M.; Chevalier C.; D’Amato D.; Desroches Y.; Duchesne F.; Gagnon L.; Ilias S.; Jerominek H.; Lagacé F.; Lambert J.; Lamontagne F.; Le Noc L.; Martel A.; Pancrati O.; Paultre J.-E.; Pope T.; Provençal F.; Topart P.; Vachon C.; Verreault S.; Bergeron A. Review of Terahertz Technology Development at INO. J. Infrared, Millimeter, Terahertz Waves 2015, 36 (10), 922–946. 10.1007/s10762-015-0181-5.

[ref23] Simoens F.; Meilhan J. Terahertz Real-Time Imaging Uncooled Array Based on Antenna- and Cavity-Coupled Bolometers. Philos. Trans. R. Soc. A 2014, 372 (2012), 20130111 10.1098/rsta.2013.0111. 24567477

[ref24] Jang D.; Kimbrue M.; Yoo Y.-J.; Kim K.-Y. Spectral Characterization of a Microbolometer Focal Plane Array at Terahertz Frequencies. IEEE Trans. Terahertz Sci. Technol. 2019, 9 (2), 150–154. 10.1109/TTHZ.2019.2893573.

[ref25] Romano M.; Chulkov A.; Sommier A.; Balageas D.; Vavilov V.; Batsale J. C.; Pradere C. Broadband Sub-Terahertz Camera Based on Photothermal Conversion and IR Thermography. J. Infrared, Millimeter, Terahertz Waves 2016, 37 (5), 448–461. 10.1007/s10762-015-0241-x.

[ref26] Oden J.; Meilhan J.; Lalanne-Dera J.; Roux J.-F.; Garet F.; Coutaz J.-L.; Simoens F. Imaging of Broadband Terahertz Beams Using an Array of Antenna-Coupled Microbolometers Operating at Room Temperature. Opt. Express 2013, 21 (4), 4817 10.1364/OE.21.004817. 23482016

[ref27] Grant J.; Escorcia-Carranza I.; Li C.; McCrindle I. J. H.; Gough J.; Cumming D. R. S. A Monolithic Resonant Terahertz Sensor Element Comprising a Metamaterial Absorber and Micro-Bolometer. Laser Photon. Rev. 2013, 7 (6), 1043–1048. 10.1002/lpor.201300087.

[ref28] Gokhale V. J.; Rais-Zadeh M. Uncooled Infrared Detectors Using Gallium Nitride on Silicon Micromechanical Resonators. J. Microelectromech. Syst. 2014, 23 (4), 803–810. 10.1109/JMEMS.2013.2292368.

[ref29] Piller M.; Luhmann N.; Chien M.-H.; Schmid S. Nanoelectromechanical Infrared Detector. Proc. SPIE 2019, 11088, 1108802 10.1117/12.2528416.

[ref30] Laurent L.; Yon J.-J.; Moulet J.-S.; Roukes M.; Duraffourg L. 12-Mm-Pitch Electromechanical Resonator for Thermal Sensing. Phys. Rev. Appl. 2018, 9 (2), 024016 10.1103/PhysRevApplied.9.024016.

[ref31] Zhang X. C.; Myers E. B.; Sader J. E.; Roukes M. L. Nanomechanical Torsional Resonators for Frequency-Shift Infrared Thermal Sensing. Nano Lett. 2013, 13 (4), 1528–1534. 10.1021/nl304687p. 23458733

[ref32] Qian Z.; Hui Y.; Liu F.; Kang S.; Kar S.; Rinaldi M. Graphene-Aluminum Nitride NEMS Resonant Infrared Detector. Microsyst. Nanoeng. 2016, 2 (1), 16026 10.1038/micronano.2016.26. 31057826 PMC6444720

[ref33] Sadeghi P.; Tanzer M.; Luhmann N.; Piller M.; Chien M.-H.; Schmid S. Thermal Transport and Frequency Response of Localized Modes on Low-Stress Nanomechanical Silicon Nitride Drums Featuring a Phononic-Band-Gap Structure. Phys. Rev. Appl. 2020, 14 (2), 024068 10.1103/PhysRevApplied.14.024068.

[ref34] Blaikie A.; Miller D.; Alemán B. J. A Fast and Sensitive Room-Temperature Graphene Nanomechanical Bolometer. Nat. Commun. 2019, 10 (1), 4726 10.1038/s41467-019-12562-2. 31624243 PMC6797740

[ref35] Zhang Y.; Hosono S.; Nagai N.; Song S.-H.; Hirakawa K. Fast and Sensitive Bolometric Terahertz Detection at Room Temperature through Thermomechanical Transduction. J. Appl. Phys. 2019, 125 (15), 151602 10.1063/1.5045256.

[ref36] Morohashi I.; Zhang Y.; Qiu B.; Irimajiri Y.; Sekine N.; Hirakawa K.; Hosako I. Rapid Scan THz Imaging Using MEMS Bolometers. J. Infrared, Millimeter, Terahertz Waves 2020, 41 (6), 675–684. 10.1007/s10762-020-00691-5.

[ref37] Belacel C.; Todorov Y.; Barbieri S.; Gacemi D.; Favero I.; Sirtori C. Optomechanical Terahertz Detection with Single Meta-Atom Resonator. Nat. Commun. 2017, 8 (1), 1578 10.1038/s41467-017-01840-6. 29146939 PMC5691196

[ref38] Calabrese A.; Gacemi D.; Jeannin M.; Suffit S.; Vasanelli A.; Sirtori C.; Todorov Y. Coulomb Forces in THz Electromechanical Meta-Atoms. Nanophotonics 2019, 8 (12), 2269–2277. 10.1515/nanoph-2019-0314.

[ref39] Fischer R.; McNally D. P.; Reetz C.; Assumpção G. G. T.; Knief T.; Lin Y.; Regal C. A. Spin Detection with a Micromechanical Trampoline: Towards Magnetic Resonance Microscopy Harnessing Cavity Optomechanics. New J. Phys. 2019, 21 (4), 043049 10.1088/1367-2630/ab117a.

[ref40] Chien M.-H.; Steurer J.; Sadeghi P.; Cazier N.; Schmid S. Nanoelectromechanical Position-Sensitive Detector with Picometer Resolution. ACS Photonics 2020, 7 (8), 2197–2203. 10.1021/acsphotonics.0c00701. 32851117 PMC7441496

[ref41] Baldacci L.; Pitanti A.; Masini L.; Arcangeli A.; Colangelo F.; Navarro-Urrios D.; Tredicucci A. Thermal Noise and Optomechanical Features in the Emission of a Membrane-Coupled Compound Cavity Laser Diode. Sci. Rep. 2016, 6 (1), 31489 10.1038/srep31489. 27538586 PMC4990904

[ref42] Ottomaniello A.; Keeley J.; Rubino P.; Li L.; Cecchini M.; Linfield E. H.; Davies A. G.; Dean P.; Pitanti A.; Tredicucci A. Optomechanical Response with Nanometer Resolution in the Self-Mixing Signal of a Terahertz Quantum Cascade Laser. Opt. Lett. 2019, 44 (23), 5663 10.1364/OL.44.005663. 31774748

[ref43] Zahl H. A.; Golay M. J. E. Pneumatic Heat Detector. Rev. Sci. Instrum. 1946, 17 (11), 511–515. 10.1063/1.1770416. 20280202

[ref44] Lang R.; Kobayashi K. External Optical Feedback Effects on Semiconductor Injection Laser Properties. IEEE J. Quantum Electron. 1980, 16 (3), 347–355. 10.1109/JQE.1980.1070479.

[ref45] Spencer P.; Rees P.; Pierce I. Theoretical Analysis. In Unlocking Dynamical Diversity; John Wiley & Sons: Chichester, U.K., 2005; pp 23–54.

[ref46] Cataldo G.; Beall J. A.; Cho H.-M.; McAndrew B.; Niemack M. D.; Wollack E. J. Infrared Dielectric Properties of Low-Stress Silicon Nitride. Opt. Lett. 2012, 37 (20), 4200 10.1364/OL.37.004200. 23073410

[ref47] Ftouni H.; Blanc C.; Tainoff D.; Fefferman A. D.; Defoort M.; Lulla K. J.; Richard J.; Collin E.; Bourgeois O. Thermal Conductivity of Silicon Nitride Membranes Is Not Sensitive to Stress. Phys. Rev. B 2015, 92 (12), 125439 10.1103/PhysRevB.92.125439.

[ref48] Mag-Isa A. E.; Jang B.; Kim J. H.; Lee H. J.; Oh C. S. Coefficient of Thermal Expansion Measurements for Freestanding Nanocrystalline Ultra-Thin Gold Films. Int. J. Precis. Eng. Manuf. 2014, 15 (1), 105–110. 10.1007/s12541-013-0311-8.

[ref49] Lugo J. M.; Oliva A. I. Thermal Properties of Metallic Films at Room Conditions by the Heating Slope. J. Thermophys. Heat Transfer 2016, 30 (2), 452–460. 10.2514/1.T4605.

[ref50] Habermehl S. Coefficient of Thermal Expansion and Biaxial Young’s Modulus in Si-Rich Silicon Nitride Thin Films. J. Vac. Sci. Technol., A 2018, 36 (2), 021517 10.1116/1.5020432.

[ref51] Hesler J. L.; Crowe T. W. NEP and Responsivity of THz Zero-Bias Schottky Diode Detectors. In 2007 Joint 32nd International Conference on Infrared and Millimeter Waves and the 15th International Conference on Terahertz Electronics; IEEE, 2007; pp 844–845.

[ref52] Ojefors E.; Pfeiffer U. R.; Lisauskas A.; Roskos H. G. A 0.65 THz Focal-Plane Array in a Quarter-Micron CMOS Process Technology. IEEE J. Solid-State Circuits 2009, 44 (7), 1968–1976. 10.1109/JSSC.2009.2021911.

[ref53] Pleteršek A.; Trontelj J. A Self-Mixing NMOS Channel-Detector Optimized for Mm-Wave and THZ Signals. J. Infrared, Millimeter, Terahertz Waves 2012, 33 (6), 615–626. 10.1007/s10762-012-9901-2.

[ref54] Bianco F.; Perenzoni D.; Convertino D.; De Bonis S. L.; Spirito D.; Perenzoni M.; Coletti C.; Vitiello M. S.; Tredicucci A. Terahertz Detection by Epitaxial-Graphene Field-Effect-Transistors on Silicon Carbide. Appl. Phys. Lett. 2015, 107 (13), 131104 10.1063/1.4932091.

[ref55] Tauk R.; Teppe F.; Boubanga S.; Coquillat D.; Knap W.; Meziani Y. M.; Gallon C.; Boeuf F.; Skotnicki T.; Fenouillet-Beranger C.; Maude D. K.; Rumyantsev S.; Shur M. S. Plasma Wave Detection of Terahertz Radiation by Silicon Field Effects Transistors: Responsivity and Noise Equivalent Power. Appl. Phys. Lett. 2006, 89 (25), 253511 10.1063/1.2410215.

[ref56] Bauer M.; Venckevičius R.; Kašalynas I.; Boppel S.; Mundt M.; Minkevičius L.; Lisauskas A.; Valušis G.; Krozer V.; Roskos H. G. Antenna-Coupled Field-Effect Transistors for Multi-Spectral Terahertz Imaging up to 425 THz. Opt. Express 2014, 22 (16), 19235 10.1364/OE.22.019235. 25321008

[ref57] Viti L.; Hu J.; Coquillat D.; Knap W.; Tredicucci A.; Politano A.; Vitiello M. S. Black Phosphorus Terahertz Photodetectors. Adv. Mater. 2015, 27 (37), 5567–5572. 10.1002/adma.201502052. 26270791

[ref58] Wang X.; Cui Y.; Li T.; Lei M.; Li J.; Wei Z. Recent Advances in the Functional 2D Photonic and Optoelectronic Devices. Adv. Opt. Mater. 2019, 7 (3), 1801274 10.1002/adom.201801274.

[ref59] Chen Y.; Ma W.; Tan C.; Luo M.; Zhou W.; Yao N.; Wang H.; Zhang L.; Xu T.; Tong T.; Zhou Y.; Xu Y.; Yu C.; Shan C.; Peng H.; Yue F.; Wang P.; Huang Z.; Hu W. Broadband Bi_2_O_2_ Se Photodetectors from Infrared to Terahertz. Adv. Funct. Mater. 2021, 31, 2009554 10.1002/adfm.202009554.

[ref60] Xu H.; Guo C.; Zhang J.; Guo W.; Kuo C.; Lue C. S.; Hu W.; Wang L.; Chen G.; Politano A.; Chen X.; Lu W. PtTe 2 -Based Type-II Dirac Semimetal and Its van Der Waals Heterostructure for Sensitive Room Temperature Terahertz Photodetection. Small 2019, 15 (52), 1903362 10.1002/smll.201903362. 31736239

[ref61] Guo C.; Guo W.; Xu H.; Zhang L.; Chen G.; D’Olimpio G.; Kuo C.-N.; Lue C. S.; Wang L.; Politano A.; Chen X.; Lu W. Ultrasensitive Ambient-Stable SnSe 2 -Based Broadband Photodetectors for Room-Temperature IR/THz Energy Conversion and Imaging. 2D Mater. 2020, 7 (3), 035026 10.1088/2053-1583/ab8ec0.

[ref62] Rogalski A.; Kopytko M.; Martyniuk P. Two-Dimensional Infrared and Terahertz Detectors: Outlook and Status. Appl. Phys. Rev. 2019, 6 (2), 021316 10.1063/1.5088578.

[ref63] Sansa M.; Sage E.; Bullard E. C.; Gély M.; Alava T.; Colinet E.; Naik A. K.; Villanueva L. G.; Duraffourg L.; Roukes M. L.; Jourdan G.; Hentz S. Frequency Fluctuations in Silicon Nanoresonators. Nat. Nanotechnol. 2016, 11 (6), 552–558. 10.1038/nnano.2016.19. 26925826 PMC4892353

[ref64] Reinhardt C.; Müller T.; Bourassa A.; Sankey J. C. Ultralow-Noise SiN Trampoline Resonators for Sensing and Optomechanics. Phys. Rev. X 2016, 6 (2), 021001 10.1103/PhysRevX.6.021001.

[ref65] Cleland A. N.; Roukes M. L. Noise Processes in Nanomechanical Resonators. J. Appl. Phys. 2002, 92 (5), 2758–2769. 10.1063/1.1499745.

[ref66] Giuliani G.; Norgia M.; Donati S.; Bosch T. Laser Diode Self-Mixing Technique for Sensing Applications. J. Opt. A Pure Appl. Opt. 2002, 4 (6), S283–S294. 10.1088/1464-4258/4/6/371.

[ref67] Song Z.; Zhang J. Achieving Broadband Absorption and Polarization Conversion with a Vanadium Dioxide Metasurface in the Same Terahertz Frequencies. Opt. Express 2020, 28 (8), 12487 10.1364/OE.391066. 32403745

[ref68] Zhang X.; Li H.; Wei Z.; Qi L. Metamaterial for Polarization-Incident Angle Independent Broadband Perfect Absorption in the Terahertz Range. Opt. Mater. Express 2017, 7 (9), 3294 10.1364/OME.7.003294.

[ref69] Tucker J. R.; Baque J. L.; Lim Y. L.; Zvyagin A. V.; Rakić A. D. Parallel Self-Mixing Imaging System Based on an Array of Vertical-Cavity Surface-Emitting Lasers. Appl. Opt. 2007, 46 (25), 6237 10.1364/AO.46.006237. 17805356

