# Supplementary material for: Micromechanical Bolometers for Subterahertz Detection at Room Temperature
Source: ACS Photonics. 2022 Jan 28;9(2):360–7. doi: 10.1021/acsphotonics.1c01273 (PMC8855436; doi:10.1021/acsphotonics.1c01273)
Supplement: Supplementary file 1 — ph1c01273_si_001.pdf [file ph1c01273_si_001.pdf]

Supporting Information:
Micromechanical bolometers for sub-Terahertz detection at room temperature.

Leonardo Vicarelli[a*], Alessandro Tredicucci[a,b*], Alessandro Pitanti[a]

[a] Laboratorio NEST, Scuola Normale Superiore and Istituto Nanoscienze - CNR, Piazza San Silvestro 12, 56127 Pisa, Italy

[b] Dipartimento di Fisica, Università di Pisa, Largo B. Pontecorvo 3, 56127 Pisa, Italy

Mechanical, thermal and optical parameters of Si_3N_4 and Cr/Au layers

The following tables provides a list of material parameters which are relevant for this work.
As explained in the Methods section of the main text, the COMSOL simulations were conducted with a single 35 nm thick Au film, rather than a 5 Cr-30 Au two-layer structure. For this reason, Table S1 contains mechanical and thermal parameters of Si_3N_4 and Au alone, while Table S2 the optical absorption of the complete Cr/Au film.
The value of the Intrinsic Stress displayed in Table S1 was not known a-priori, and was therefore adjusted to match simulated and experimental resonance frequency of the membranes.
The radiation absorptions reported in Table S2 were calculated from the real and imaginary parts of the refractive indices of the materials. Si_3N_4 was assumed to be completely transparent for all frequencies under consideration[1].

	Si_3N_4	Au
Thickness (nm)	300	35
Density (g cm^{-3})	3,1	19,3
Thermal expansion coefficient α^{-1}	1.5x10^{-6} ([2])	10.0x10^{-6} ([3])
Specific heat (J kg^{-1} K^{-1})	800 ([4])	125 ([5])
Thermal conductivity (W m^{-1} K^{-1})	3 ([4])	152 ([5])
Young's modulus (GPa)	260 ([6])	90([7])
Poisson's ratio	0.28 ([8])	0.42 ([7])
Intrinsic Stress (MPa)	925	--

Table S 1 Empirical material parameters used in the COMSOL simulation.

	Si_3N_4	Cr/Au
Sub-THz (f=0.14 THz) ([9])	Transparent	14%
Infrared (λ=945 nm) ([10])	Transparent	3,3%
Green Laser (λ=532 nm) ([10])	Transparent	17%

Table S 2 Calculated radiation absorption used in the COMSOL simulation.

Calculation of thermal conductance, heat capacity and total mass

Using the material parameters of Table S1, we calculated the individual contribution of the Si_3N_4 and the Au layers to the thermal conductance, heat capacity and total mass of both devices. It clearly emerges how the largest contribution to heat dissipation is given by the metal layer, whereas the Si_3N_4 layer mostly provides thermal mass to the device. The ratio between the thermal conductance and the heat capacity gives an approximate value for the thermal response frequency of the devices, resulting in f_{M1}~12 Hz and f_{M2}~22 Hz.

**Device M1**	Si_3N_4 (300 nm)	Au (35 nm)	Si_3N_4 + Au
Thermal conductance of the 4 tethers (W K^{-1})	0,17 x10^{-6}	1,00 x10^{-6}	1,17 x10^{-6}
Heat capacity of the central plate (J K^{-1})	8,30 x10^{-8}	0,94 x10^{-8}	9,24 x10^{-8}
Total mass (kg)	1,38x10^{-10}	1,00 x10^{-10}	2,38x10^{-10}
**Device M2**			
Thermal conductance of the 4 tethers (W K^{-1})	0,14 x10^{-6}	0,82 x10^{-6}	0,96 x10^{-6}
Heat capacity of the central plate (J K^{-1})	3,85 x10^{-8}	0,44 x10^{-8}	4,29 x10^{-8}
Total mass (kg)	0,90 x10^{-10}	0,65 x10^{-10}	1,55 x10^{-10}

Table S 3 Individual contribution of Si_3N_4 and Au layers to the thermal conductance and heat capacity of devices M1 and M2

Infrared laser responsivity for device M2

The following figure shows the linear responsivity of device M2, as function of the impinging infrared laser power, similarly to Figure 2 of the main text. As stated in the main text, the responsivity is 187 kHz/W. Given the resonance frequency at zero incident power f_0=91.26 kHz, the normalized responsivity is 2.05 W^{-1}.

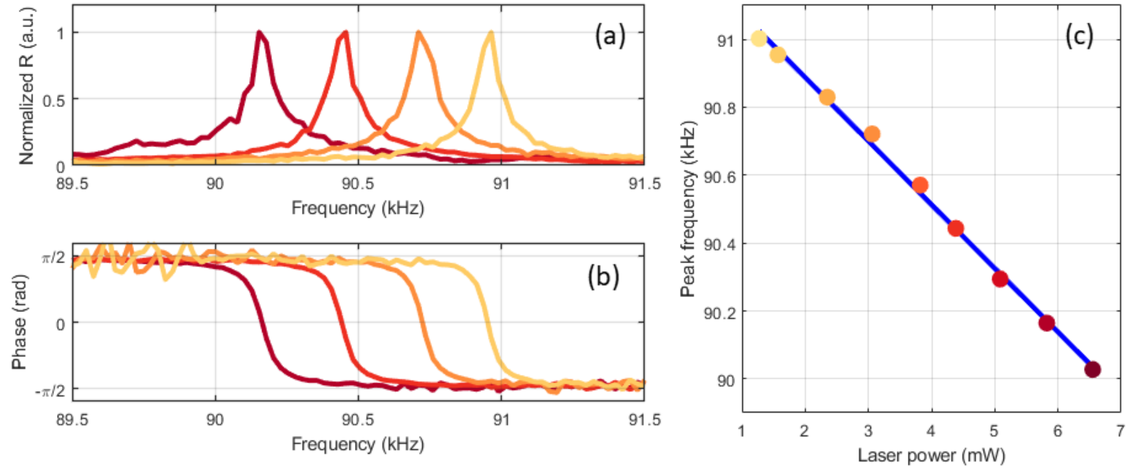

Figure S 1 Device M2. Demodulated amplitude (a) and phase (b) when probing the trampoline resonator at different laser powers of the infrared laser, used for the self-mixing readout. (c) Shift of the resonant frequency as a function of laser power.

Infrared laser responsivity for device M2 without Cr/Au metal layer

The following figure shows responsivity of device M2, measured before the deposition of the Cr/Au metal layer, as function of the impinging infrared laser power. Here, the responsivity is 8 kHz/W, approximately 23 times smaller than the responsivity after metal deposition.

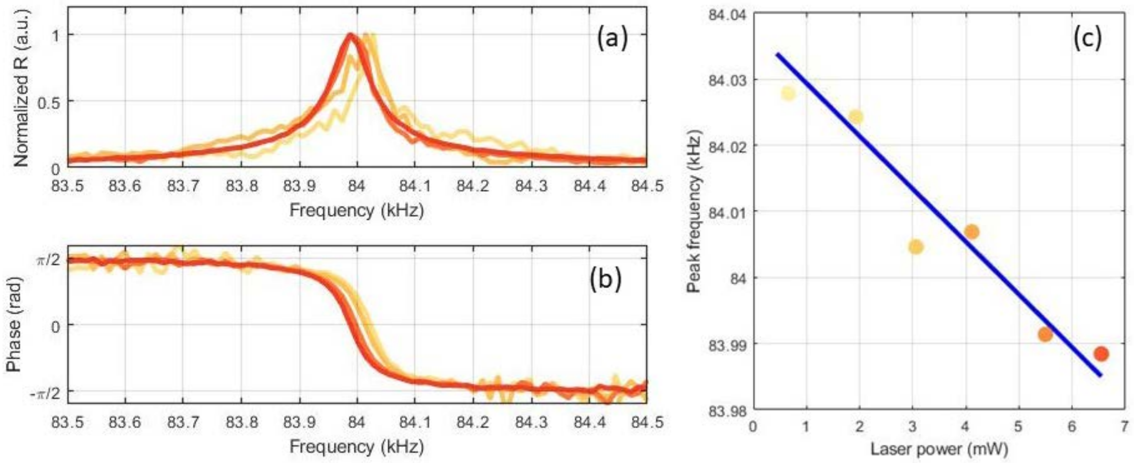

Figure S 2 Device M2, before the Cr/Au layer deposition. Demodulated amplitude (a) and phase (b) when probing the trampoline resonator at different laser powers of the infrared laser, used for the self-mixing readout. (c) Shift of the resonant frequency as a function of laser power.

Green laser responsivity

The linearity of device responsivity was also tested using the green laser (532 nm), adding Neutral Density filters with increasing opacity directly in front of the source. The shift of the resonance peak was measured similarly to the infrared case, sweeping the excitation frequency sent the piezo actuator and reading the self-mixing signal via a single lock-in channel. The infrared laser used as probe for the self-mixing readout was kept slightly above lasing threshold (1.5 mW) during the measurement. The obtained green laser responsivities are 520 kHz/W and 600 kHz/W for device M1 and M2, respectively. The latter value (M2) is compatible with the measurement of phase responsivity described in the main text.

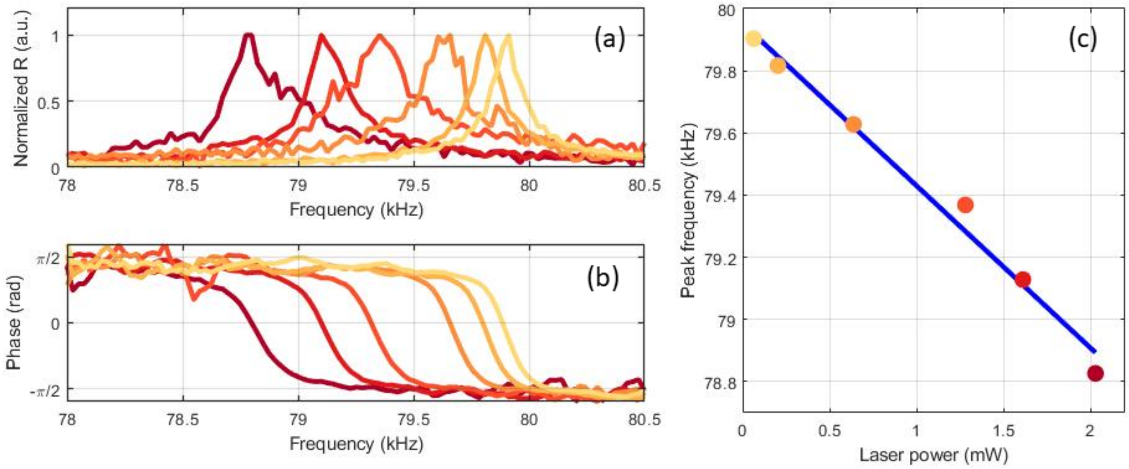

Figure S 3: Device M1. Demodulated amplitude (a) and phase (b) when probing the trampoline resonator at different laser powers of the green laser. (c) Shift of the resonant frequency as a function of laser power.

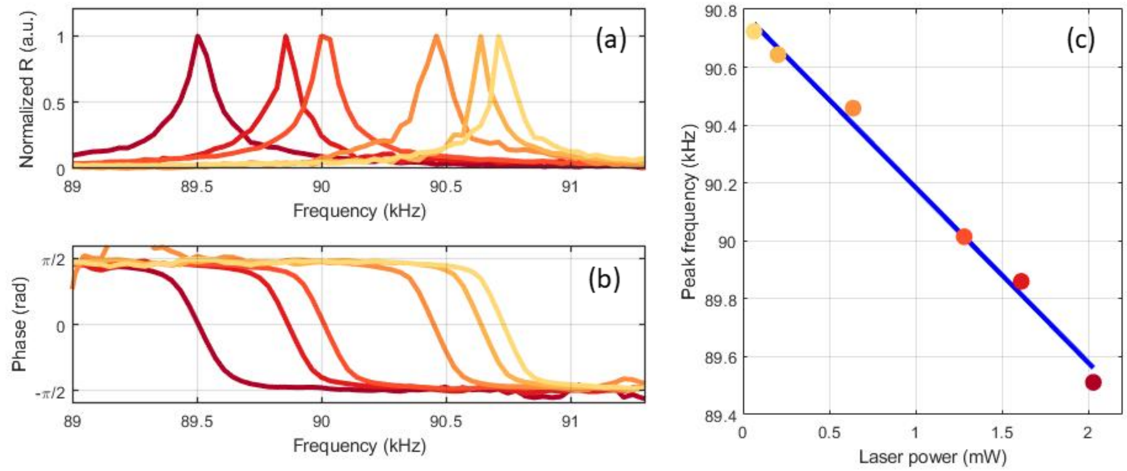

Figure S 4: Device M2. Demodulated amplitude (a) and phase (b) when probing the trampoline resonator at different laser powers of the green laser. (c) Shift of the resonant frequency as a function of laser power.

Spot size measurement of the sub-THz source

We measured the spot size of the sub-THz source, after the reflection in the parabolic mirror, mapping the 2D intensity of the beam with a calibrated pyrometer. The map was then fitted with a 2D Gaussian profile, convoluted with the active area of the pyrometer, given by a disk of radius 5 mm. The beam shape is very asymmetric in the membrane plane, elongated along the x-direction, because of the reflection in the parabolic mirror.

In particular, $2\sigma_x$= 7 .1 mm and $2\sigma_y$= 4.3 mm.

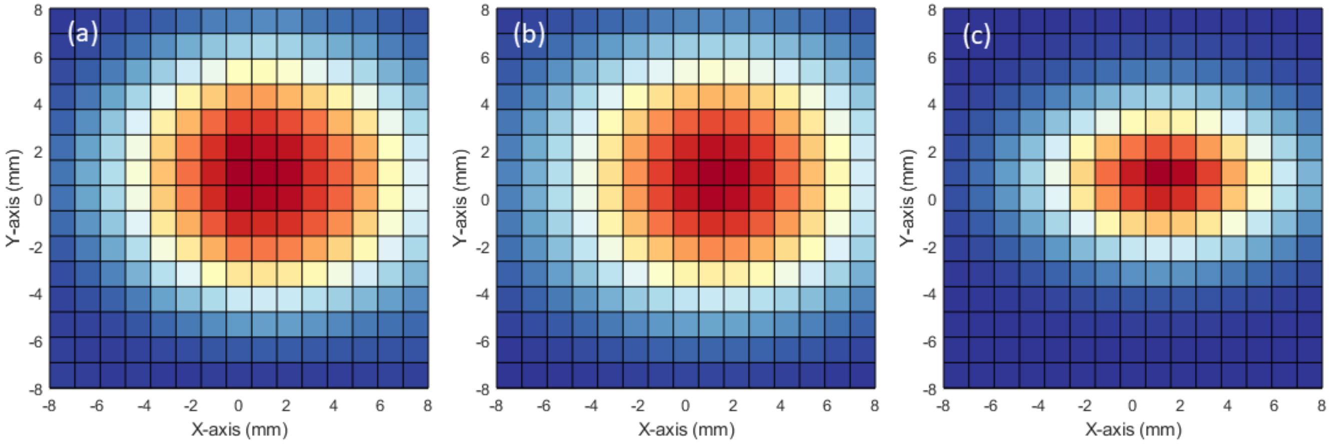

Figure S 5: Measurements and fitted 2D maps of the sub-THz beam. (a) Measured map. (b) Fitted map, convoluted with a 5 mm radius disk. (c) Deconvolution of the fitted map, showing the actual beam profile.

Spot size measurements of green and infrared lasers

We measured the spot size of both green and infrared laser (intended as twice the standard deviation 2σ of a Gaussian beam profile) using the knife edge method.

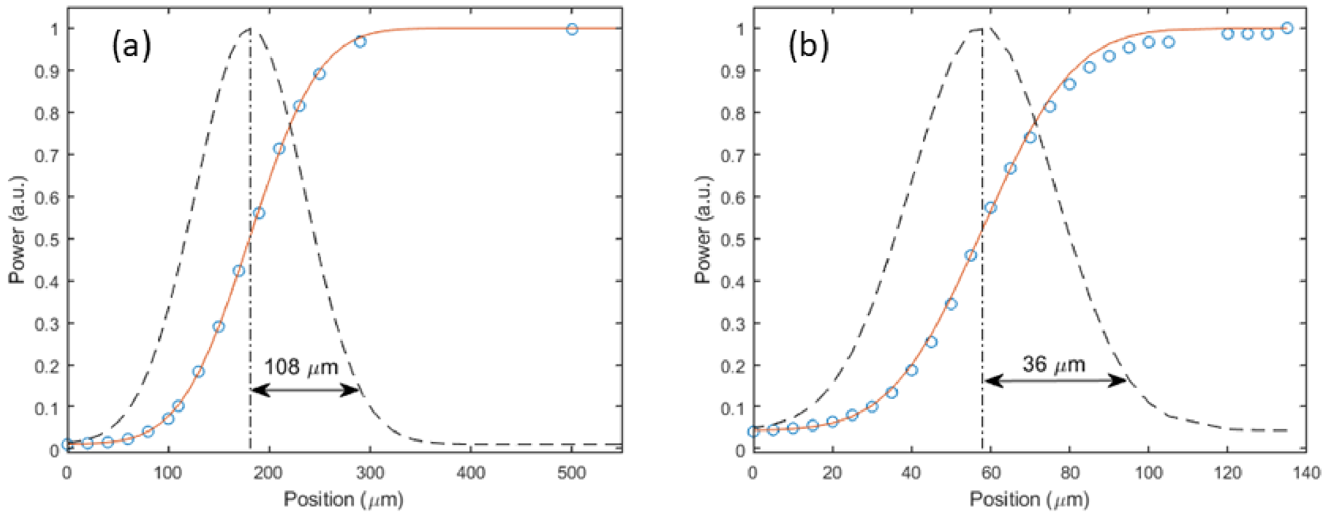

Figure S 6 Measurements of the spot sizes (2σ) of the green laser (a) and the infrared laser (b) by the knife-edge method.

Self-mixing signal amplitude as function of the piezo actuator voltage

We verified the linear response of the self-mixing signal, read after the lock-in amplifier, as function of the piezo driving voltage. The following figure refers to the measurements performed on device M1.

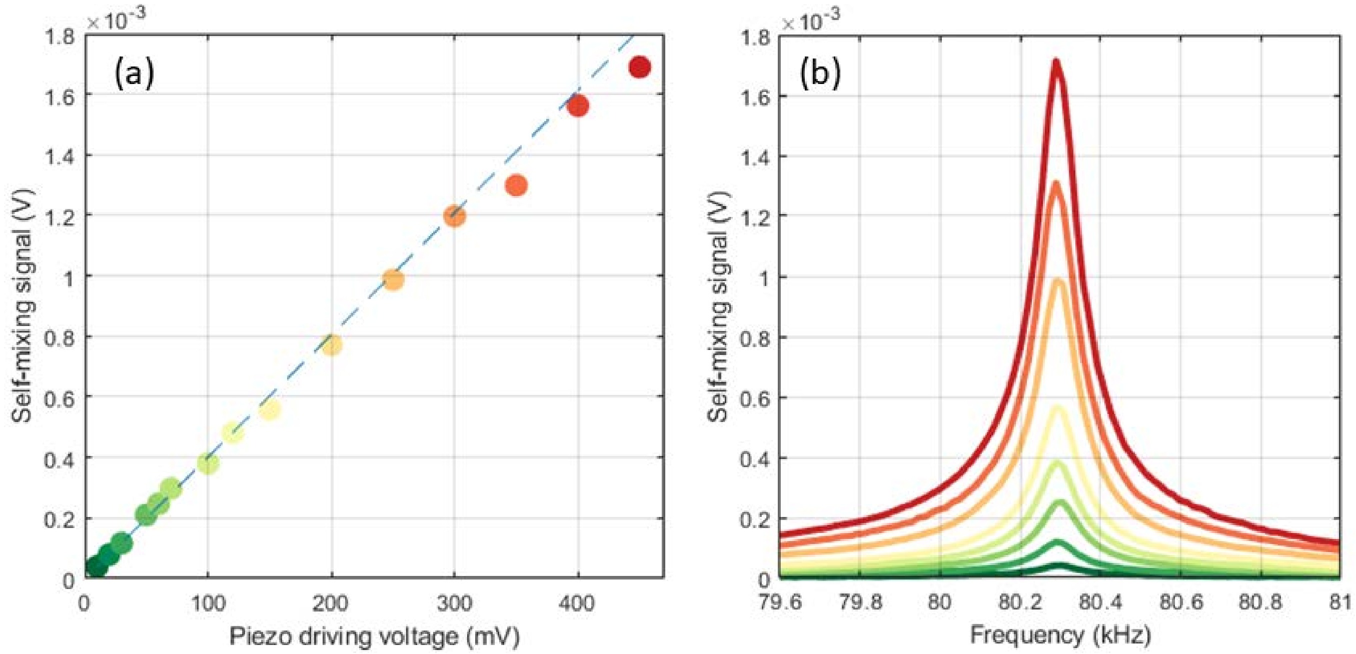

Figure S 7: (a) Intensity of the self-mixing signal, read at the lock-in output, as function of the piezo driving voltage amplitude. (b) Lorentzian curves obtained sweeping the driving voltage frequency around the resonance point, with increasing piezo driving voltages.

REFERENCES

(1) Cataldo, G.; Beall, J. A.; Cho, H.-M.; McAndrew, B.; Niemack, M. D.; Wollack, E. J. Infrared Dielectric Properties of Low-Stress Silicon Nitride. *Opt. Lett.* 2012, *37* (20), 4200.

(2) Habermehl, S. Coefficient of Thermal Expansion and Biaxial Young's Modulus in Si-Rich Silicon Nitride Thin Films. *J. Vac. Sci. Technol. A Vacuum, Surfaces, Film.* 2018, *36* (2), 021517.

(3) Mag-Isa, A. E.; Jang, B.; Kim, J. H.; Lee, H. J.; Oh, C. S. Coefficient of Thermal Expansion Measurements for Freestanding Nanocrystalline Ultra-Thin Gold Films. *Int. J. Precis. Eng. Manuf.* 2014, *15* (1), 105–110.

(4) Ftouni, H.; Blanc, C.; Tainoff, D.; Fefferman, A. D.; Defoort, M.; Lulla, K. J.; Richard, J.; Collin, E.; Bourgeois, O. Thermal Conductivity of Silicon Nitride Membranes Is Not Sensitive to Stress. *Phys. Rev. B* 2015, *92* (12), 125439.

(5) Lugo, J. M.; Oliva, A. I. Thermal Properties of Metallic Films at Room Conditions by the Heating Slope. *J. Thermophys. Heat Transf.* 2016, *30* (2), 452–460.

(6) Chuang, W.-H.; Luger, T.; Fettig, R. K.; Ghodssi, R. Mechanical Property Characterization of LPCVD Silicon Nitride Thin Films at Cryogenic Temperatures. *J. Microelectromechanical Syst.* 2004, *13* (5), 870–879.

(7) Faurie, D.; Renault, P.-O.; Le Bourhis, E.; Villain, P.; Goudeau, P.; Badawi, F. Measurement of Thin Film Elastic Constants by X-Ray Diffraction. *Thin Solid Films* 2004, *469–470* (SPEC. ISS.), 201–205.

(8) Vlassak, J. J.; Nix, W. D. A New Bulge Test Technique for the Determination of Young's Modulus and Poisson's Ratio of Thin Films. *J. Mater. Res.* 1992, *7* (12), 3242–3249.

(9) Zhou, D.; Parrott, E. P. J.; Paul, D. J.; Zeitler, J. A. Determination of Complex Refractive Index of Thin Metal Films from Terahertz Time-Domain Spectroscopy. *J. Appl. Phys.* 2008, *104* (5), 053110.

(10) Windt, D. L. IMD—Software for Modeling the Optical Properties of Multilayer Films. *Comput. Phys.* 1998, *12* (4), 360.
